# Nutrient-Dense African Indigenous Vegetables and Grains in the FAO Food Composition Table for Western Africa (WAFCT) Identified Using Nutrient-Rich Food (NRF) Scores

**DOI:** 10.3390/nu16172985

**Published:** 2024-09-04

**Authors:** Jonathan Lara-Arevalo, Amos Laar, M. Pia Chaparro, Adam Drewnowski

**Affiliations:** 1 Department of Nutrition, Gillings School of Global Public Health, University of North Carolina at Chapel Hill, Chapel Hill, NC 27599, USA; jonathan.lara@unc.edu; 2 Department of Population, Family and Reproductive Health, School of Public Health, University of Ghana, Legon, Accra P.O. Box LG 25, Ghana; alaar@ug.edu.gh; 3 Food Systems, Nutrition, and Health Program, School of Public Health, University of Washington, Seattle, WA 98195, USA; pchap@uw.edu; 4 Department of Health Systems and Population Health, School of Public Health, University of Washington, Seattle, WA 98195, USA; 5 Center for Public Health Nutrition, University of Washington, Seattle, WA 98195, USA

**Keywords:** nutrient profiling, nutrient-rich foods (NRF index), priority micronutrients, African indigenous vegetables, African indigenous grains, animal-source foods

## Abstract

Nutrient profiling (NP) models that assess nutrient density of foods can help remedy micronutrient deficiencies. We used NP methods to identify the most nutrient-rich foods in the 2019 FAO/INFOODS Western Africa Food Composition Table (WAFCT). The WAFCT had complete data for 909 foods. Three versions of the well-established Nutrient-Rich Food (NRF) model were used: (1) the original NRF9.3 based on 9 micronutrients (protein, fiber, calcium, iron, potassium, magnesium, vitamin A, vitamin C, and vitamin D); (2) a new NRF6.3 based on 6 priority micronutrients (iron, zinc, calcium, folate, vitamin A, vitamin B12); and (3) NRF 15.3, based on 15 nutrients to encourage (NRF6.3 nutrients + vitamin D, vitamin E, vitamin C, vitamin B1, vitamin B2, vitamin B3, vitamin B6, copper, and magnesium). Data analyses used one-way ANOVAs and independent *t*-tests, with significance at α = 0.05. Animal-source foods were rated higher by NRF6.3 priority micronutrient and NRF15.3 NP models than by the NRF9.3 model. African indigenous vegetables had higher protein content and higher nutrient density compared to non-indigenous vegetables, and African indigenous grains had higher nutrient density compared to non-indigenous grains. Though animal-source foods received some of the highest scores, NP models adapted to the West African context showed that African indigenous vegetables and grains were also nutrient rich. Indigenous foods could be important sources of priority micronutrients for the region.

## 1. Introduction

Starchy diets commonly consumed in Sub-Saharan Africa often lack various micronutrients, including iron, zinc, calcium, folate, iodine, vitamin A, and vitamin B12 [1,2,3]. One reason for micronutrient deficits might be limited dietary diversity [4], a proxy measure for micronutrient adequacy [5,6]. Studies using the United Nations Food and Agriculture Organization (FAO) Minimum Dietary Diversity Score for Women (MDDW) [7] showed that increasing dietary diversity can prevent multiple micronutrient deficiencies [8,9]. Regional dietary guidelines for West Africa have likewise stressed the importance of diverse diets, promoting the consumption of grains, vegetables, and fruit along with animal products such as eggs, dairy, fish, and organ meats [10]. However, to date, only 4 (i.e., Benin, Ghana, Nigeria, and Sierra Leone) out of the 16 countries in Western Africa have developed food-based dietary guidelines [11,12,13,14]. 

Nutrient profiling (NP) methods can help in the implementation of food-based dietary guidelines. NP methods are designed to identify nutrient-rich foods that best fulfil population nutrient requirements and address identified health needs [15]. For example, the WHO African Region NP model, designed to prevent childhood obesity, penalizes foods that are high in calories, saturated fat, added sugar, and salt but do not include any micronutrients at all [16]. While many NP models are based on nutrients that should be limited (e.g., fat, sugar, salt), they can also include nutrients to encourage (e.g., protein, fiber, vitamins, and minerals) or some combination of both. NP models that include micronutrients might be the best option for West Africa, where micronutrient deficiencies remain a significant concern.

New NP models, sensitive to local contexts and health needs, need to be based on local and regional nutrient composition databases [17,18]. This study applied variants of the well-established Nutrient-Rich Food Index (NRF) [19] to regional foods in the Food Composition Table for Western Africa (WAFCT 2019) [20], maintained by the FAO and the International Network of Food Data Systems (INFOODS). The WAFCT contains nutrient values for more than 1000 foods frequently consumed in this region [20]. 

Our goal was to identify the most nutrient-rich foods in the WAFCT, using three different NRF models. We sought to investigate whether foods rich in the region’s priority micronutrients could be identified among African indigenous vegetables and grains.

## 2. Materials and Methods

### 2.1. Nutrient Composition Database

The FAO/INFOODS Food Composition Table for Western Africa 2019 (WAFCT) [20] lists 1028 foods and beverages, their names in English and French, along with energy and nutrient content. The original WAFCT had multiple missing nutrient values. Where possible, nutrient values for the same raw foods were obtained from the USDA Food and Nutrient Database for Dietary Studies 2017–2018 [21]. Foods with missing values that could not be matched were excluded. Values for added sugars were imputed using standard procedures [22]. For mixed dishes, added sugars for each ingredient were calculated and summed, following WAFCT recipes provided. After excluding 119 items, the 909 foods with complete information were analyzed using the WAFCT 14 food categories. The categories were cereals (18.6%), meat and poultry (13%), legumes (12.7%), vegetables (12.4%), fish and seafood (11.7%), starchy roots and tubers (9.8%), fruits (4.8%), fats and oils (3.9%), soups and sauces (3.7%), nuts and seeds (3.3%), milk and dairy (2.5%), eggs (1.5%), beverages (1.3%), and miscellaneous foods (0.8%). Appendix A provides examples for each food category.

The present analyses added categories to align with the FAO Minimum Dietary Diversity for Women Indicator [7]. Vegetables (*n* = 113) were divided into dark green leafy vegetables (52.2%), vitamin A-rich vegetables (10.6%), and other vegetables (37.2%). Fruits (*n* = 44) were divided into vitamin A-rich fruits (18.2%) and other fruits (81.8%).

Vegetables and grains in WAFCT were further classified into African indigenous vegetables (AIVs, *n* = 43) and African indigenous grains (AIGs, *n* = 55). Vegetables were classified as AIV if they were in their raw or minimally processed state, or as AIV preparations if they were incorporated into mixed meals or recipes. There were 10 basic AIV items: amaranth leaves, spider plant, jute mallow, cowpea leaves, native eggplant, pumpkin leaves, moringa, sweet potato leaves, okra, and okra leaves. AIGs were raw or minimally processed grains, as well as flours derived from these grains. There were 5 types of AIGs: fonio, pearl millet, teff, sorghum, and native rice. Non-indigenous grains included maize, wheat, rice, and oats. Mixed dishes using indigenous and non-indigenous grains were excluded from the AIG analyses.

Foods were manually classified into animal-source or plant-source foods based on protein content. Animal-source foods included meat and poultry protein; milk, eggs, and dairy protein; and fish and seafood protein. For mixed dishes, recipe ingredients were used to determine the percentage of protein of each of the types in the dish. For nutrient profiling, food items with energy density of <10 kcal/g, such as water, diet beverages, and coffee and tea were excluded. Also excluded were alcoholic beverages, herbs, and spices.

### 2.2. Nutrient-Rich Food Indices

The Nutrient-Rich Food Index (NRF) has two subscores that are based on a variable number of nutrients to encourage (*NRn*) (Equation (1)) and nutrients to limit (Equation (2)) [19,23]. This study used three variants of the NRF: NRF9.3 (standard version), NRF6.3 (priority micronutrients), and NRF15.3 (extended priority nutrients). NRF9.3 includes nine nutrients to encourage, including protein, fiber, calcium, iron, potassium, magnesium, vitamin A, vitamin C, and vitamin D. The NRF6.3 Priority Micronutrients score adds 6 further nutrients to encourage commonly lacking in the West African region [3]: iron, zinc, calcium, vitamin A (RAE), vitamin B12, and folate. The NRF15.3 Extended Priority Nutrients score adds 15 additional nutrients to encourage: vitamin A, vitamin D, vitamin E, vitamin C, vitamin B1, vitamin B2, vitamin B3, vitamin B6, vitamin B12, folate, copper, calcium, iron, magnesium, and zinc. All three models use the same three nutrients to limit (LIM): sodium, saturated fats, and added sugars. 

Nutrient standards were taken from the *Codex Alimentarius* [24] (Table 1). The final *NRF_n.k_* scores (Equation (3)) are calculated as the sum of percent daily values for *n* nutrients to encourage (*Nut_inc_i_*) minus the sum of percent daily values for the 3 nutrients to limit (*Nut_lim_i_*). Percent daily values (%*DV*) were calculated per 100 kcal (energy density; *ED*) of food and were capped at 100%; the mathematical expressions are given below.

(1) $$NRn=\left(\frac{Nut\_inc_{1}}{DV_{1}}+\frac{Nut\_inc_{2}}{DV_{2}}+\ldots +\frac{Nut\_inc_{i}}{DV_{n}}\right)\times (100/ED)$$

(2) $$LIMk=\left(\frac{Nut\_lim_{1}}{MRV_{1}}+\ldots +\frac{Nut\_lim_{k}}{MRV_{k}}\right)\times (100/ED)$$

(3) $$NRFn.k=\left[\left.\sum_{i=1}^{n}\frac{Nut\_inc_{i}}{\left(DV_{i}\right)}-\sum_{j=1}^{k}\frac{Nut\_lim_{k}}{\left(MRV_{j}\right)}\right]\right.\times (100/ED)$$

### 2.3. Statistical Analysis

Mean, standard deviation, and median scores of NRF9.3, NRF6.3, and NRF15.3 were calculated for each food group. Sensitivity analyses were conducted to evaluate the robustness of the findings, with and without the inclusion of outliers. 

One-way ANOVAs and independent *t*-tests were used to conduct multiple comparisons of nutrient density and protein content across food groups. The Bonferroni correction was used to adjust for multiple testing, and any significant differences were indicated. The significance level for all tests was set at α = 0.05. SPSS 28 software (IBM, Armonk, NY, USA) was used to perform all statistical analyses.

## 3. Results

### 3.1. Nutrient Density

**Figure 1:**
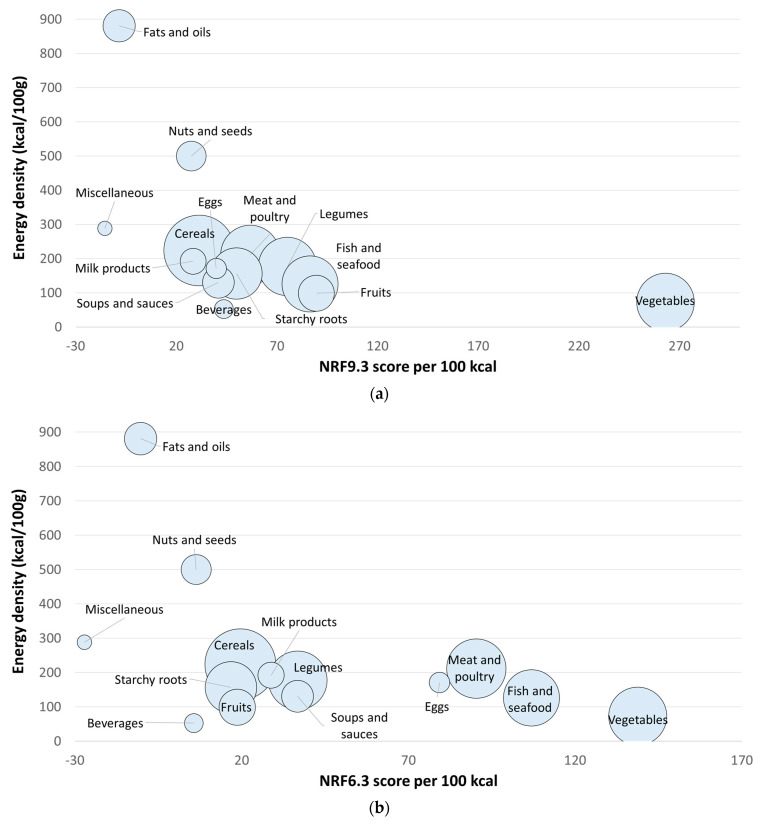

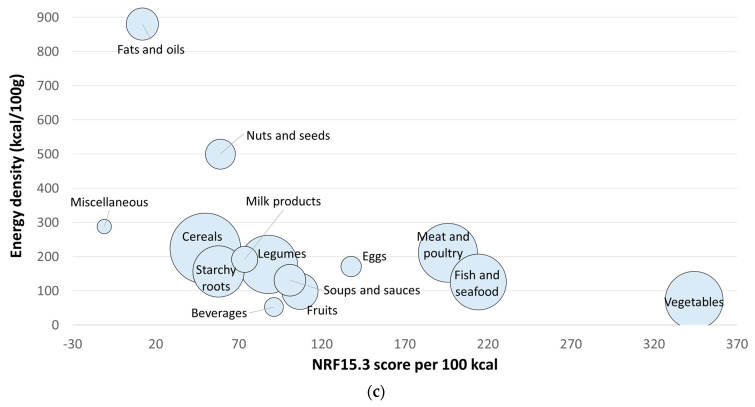
Nutrient-Rich Foods Index scores per 100 kcal. for each WAFCT food category plotted against energy density per 100 g. (**a**) NRF9.3; (**b**) NRF6.3 for priority micronutrients; (**c**) NRF15.3 extended priority nutrients. The size of the circles represents the number of foods in each food category.

Table 2 shows means, standard deviations, and medians for NRF9.3 by the 14 WAFCT food groups. Based on NRF9.3, the vegetable group (263) had the highest score of all food groups, having a significant difference to the next highest food group score (fruits: 90). Fish and seafood, and legumes were the next food groups with the highest scores. Fats and oils, and miscellaneous foods (containing mostly sugars and marmalades) were the food groups with the lowest NRF9.3 scores. Figure 1a shows the distribution of NRF9.3 scores and energy density by WAFCT food group.

Table 3 shows scores of the NRF6.3 by the 14 WAFCT food groups. Vegetables were the food group with the highest NRF6.3 score, followed by fish and seafood, meat and poultry, eggs, and legumes. Fruits were found to be in the ninth place out of the 14 food groups based on NRF6.3. Moreover, miscellaneous foods, fats and oils, and beverages were the food groups with the lowest NRF6.3 scores. Figure 1b shows the distribution of NRF6.3 (priority micronutrients) scores and energy density by WAFCT food group.

Table 4 shows mean, standard deviation, and median scores of the NRF15.3 (extended priority nutrients) by the 14 WAFCT food groups. Based on the NRF15.3 scores, the ranking of food groups also changed. Since more nutrients were used in the model, the overall scores were higher in value. Like the previous NRF versions, vegetables were the food group with the highest NRF15.3 scores. Moreover, animal-source foods received some of the highest NRF15.3 scores, compared to the NRF9.3. Fish and seafood was the second food group with highest scores, just as with the NRF6.3. The next food groups with the highest scores were meat and poultry, eggs, fruits, and soups and sauces. Figure 1c shows the distribution of NRF15.3 (extended priority nutrients) scores and energy density by WAFCT food group. 

Table 5 shows a list of the top 20 foods with the highest scores for each of the NRF versions. Dark green leafy vegetables, both raw and boiled, lead all lists, as they were the most nutrient-dense foods. However, while the top NRF9.3 foods are only leafy vegetables, the top NRF6.3 and NRF15.3 lists also contain poultry, meat, fish, and organ foods.

#### 3.1.1. Nutrient Density of African Indigenous Vegetables (AIVs)

Nutrient density and protein content of AIVs and mixed AIV dishes were analyzed and compared with the rest of the vegetables. Table 6 shows that all three NRF models gave higher nutrient density scores to AIVs than to non-indigenous vegetables; the differences were more pronounced with the NRF6.3 and NRF15.3 (better adapted to West African needs), than with NRF9.3. The predominance of AIVs in the top 20 NRF scores list can also be observed in Table 5. Protein content of mixed dishes was also assessed. Overall, preparations with AIVs had more protein mainly because various local AIV preparations also contained some fish and meat. 

#### 3.1.2. Nutrient Density of African Indigenous Grains (AIG)

AIG were compared to non-indigenous grains using different NRF models as shown in Table 6. AIGs scored higher than non-indigenous grains; however, only the NRF9.3 scores were significantly different. After removing 10 fortified flours, comparing AIGs and non-fortified non-indigenous grains showed that the three NRF scores were significantly higher for AIG compared to non-indigenous grains.

## 4. Discussion

The present study applied NP methods to evaluate nutrient density of more than 900 foods in the FAO West Africa database. The NP models were adapted to the West Africa context, taking differences in micronutrient nutrition into account. For instance, while the United States identifies dietary fiber, calcium, potassium, iron, and vitamin D as nutrients of public health concern [25], LMICs commonly face deficiencies in iron, zinc, folate, vitamin A, calcium, and vitamin B12 [3]. Many existing nutrient profiling tools, including the NRF, may need to be adapted for use in LMICs by incorporating priority micronutrients of interest [17,18].

Three NRF versions were used to assess nutrient density of West Africa foods. Only the original NRF9.3 model included protein and fiber as nutrients to encourage; the other NP models did not. The NRF6.3 model featured priority micronutrients known to be missing from some overly starchy diets consumed in West Africa. These were iron, zinc, calcium, folate, vitamin A, vitamin B12. The more comprehensive NRF15.3 score included vitamin A, vitamin D, vitamin E, vitamin C, vitamin B1, vitamin B2, vitamin B3, vitamin B6, vitamin B12, folate, copper, calcium, iron, magnesium, and zinc. 

All three NRF models rated vegetables as the most nutrient rich food group. Within the vegetable group, dark green leafy vegetables had the highest nutrient density scores, followed by vitamin A-rich vegetables. The FAO Minimum Dietary Diversity for Women (MDD-W) specifically features dark green leafy vegetables and vitamin A-rich vegetables as important sources of micronutrients [7]. The MDD-W food groups represent the main sources of priority micronutrients that are essential for women of childbearing age. Moreover, dietary diversity is a proxy measure for micronutrient adequacy [5,6]. 

Based on NRF scores, African indigenous vegetables (AIVs) had higher nutrient density and higher protein content compared to non-indigenous vegetables. Various AIVs were also classified as dark green leafy vegetables. Similarly, African indigenous grains (AIGs) presented substantially higher nutrient density scores than non-fortified non-indigenous grains. The enhanced nutritional content of indigenous vegetables and grains may be attributed to their reduced need for fertigation, better adaptation to local soils, and lower requirements for nutrients and water [26]. These findings emphasize the vital role of African indigenous vegetables and grains in providing essential micronutrients. Thus, advocating for the consumption of local foods should be prioritized when developing strategies and programs aimed at addressing malnutrition in the Western Africa region. Other studies have also identified AIVs as a potential solution to malnutrition [27] and even as climate resilient foods due to their tolerance to high temperatures and precipitation [28]. However, various challenges, such as inadequate progress in agronomic techniques and limited access to AIVs in the marketplace, have been identified [29]. An increase in the consumption of AIVs could improve micronutrient deficiencies within at-risk populations in Western Africa.

West African diets can be high in carbohydrate content, incorporating grains, cereals, legumes, roots, tubers, and plantains [30]. The present analyses pointed to specific AIGs as important sources of micronutrients. The nutritional advantages of AIGs have been previously documented [31], and more recent evidence indicates a growing interest in these foods [32,33,34].

Micronutrient-centered NRF6.3 and NRF15.3 models gave higher ratings to animal-source foods compared to plant foods, even though protein was not a component of those two NP models. Animal proteins are rich in multiple priority micronutrients and can play a pivotal role in LMIC’s nutrition [35]. The FAO has emphasized the importance of meat, eggs, and milk as critical sources of vital nutrients that are not readily obtainable from plant-based food sources [36]. The significance of fish and seafood has also been underscored [37]. However, livestock, including both meat and dairy, has been linked to higher greenhouse gas emissions and a negative planetary impact [38]. Consequently, there has been a push to reduce the consumption of animal-source food to mitigate the impacts of climate change [39,40,41]. While reducing meat consumption is advocated as a means of improving health and reducing the impacts of climate change, most of the evidence comes from high income countries that may already be at peak meat consumption [38,42]. That is not the case across most LMICs. Priority micronutrients in LMICs are predominantly found in organs, meats, dark green leafy vegetables, seafood, and eggs [3]. Higher consumption of animal-source foods can lead to improved nutrient intake and reduced malnutrition among many populations in sub-Saharan Africa [35]. Moreover, reducing meat consumption in LMICs could adversely affect the livelihoods of many low-income populations who rely on livestock, poultry, and fishing [43]. Therefore, dietary recommendations in LMICs should carefully consider these trade-offs. 

Our study had some limitations: First, having access to accurate local nutrient composition data is the first step in NP modeling [17]. The WAFCT [20] had to be updated, revised, and checked for missing data before it could be used as proxy for the regional food supply. The published WAFCT still had a significant number of missing nutrient values that needed to be cross referenced with other data and/or imputed based on the existing literature. Data from the USDA were utilized to fill the gaps in nutrient composition; however, nutrient profiles of foods may differ across different regions [44]. Significant effort went into quality control, leading to the development of a cleaned and coded WAFCT database for 909 items. Second, the WAFCT database primarily consists of minimally processed foods along with some processed foods. Not all foods within a given food group are of equivalent nutritional value. Nutrient profiling models can help to identify and rank locally available nutrient-rich foods within each food group [45]. There is a need for a comprehensive database of branded processed and ultra-processed foods in the West Africa region that has both nutrient content and an electronic ingredient list [46]. The WAFCT may no longer represent the totality of the West Africa food supply and does not include many of the packaged processed foods currently consumed by the population. Moreover, iodine deficiency remains a global health concern, particularly in low- and middle-income countries. Unfortunately, the WAFCT lacks information on iodine content, impeding its integration into the NRF models. Lastly, micronutrient deficiencies often stem from the high cost of a nutrient-rich diet [47,48,49]. High rates of micronutrient deficiencies in the Western Africa region [50] can be remedied by improved access to nutrient-rich and affordable foods [51]. We were not able to assess food costs due to the absence of food price data in West Africa.

## 5. Conclusions

To the best of our knowledge, the present analyses represent the first application of NP methods to the WAFCT 2019 dataset. Demonstrating high nutrient density of African indigenous vegetables (AIVs) and African indigenous grains (AIGs) was a finding of particular interest. Despite encountering some challenges, such as gaps in nutrient data and the absence of comprehensive information on ultra-processed foods, our findings provide valuable insights into the potential of indigenous foods to enhance diet quality in West Africa. Local agriculture has the potential to improve the population’s diet quality by supplying nutrient rich foods at an affordable cost. Therefore, efforts should be made to promote this agriculture and to integrate indigenous foods into the region’s food-based dietary guidelines.

## Figures and Tables

**Table 1:** Reference daily values (DVs) for micronutrients of interest.

Nutrients to Encourage	Reference Daily Values *
Protein (g)	50
Fiber (g)	25
Vitamin A (RAE)	800
Vitamin C (mg)	100
Vitamin D (mcg)	15
Calcium (mg)	1000
Iron (mg)	14
Potassium (mg)	3500
Magnesium (mg)	310
Zinc (mg)	11
Folate (mcg)	400
Copper (mcg)	900
Vitamin B1 (mg)	1.2
Vitamin B2 (mg)	1.2
Vitamin B3 (mg NE)	15
Vitamin B6 (mg)	1.3
Vitamin B12 (mcg)	2.4
Nutrients to limit	Maximum recommended values
Saturated fat (g)	20
Added sugars (g)	50
Sodium (mg)	2000

* Daily values were taken from the *Codex Alimentarius* [24].

**Table 2:** NRF9.3 scores by WAFCT food group per 100 kcal.

WAFCT Food Groups	No. of Foods	Kcal/100 g	NRF9.3 Scores		
			Mean ^1^	SD	Median
Vegetables	113	73	263 ^a^	123	234
*Dark green leafy vegetables*	59	62	344	105	360
*Other vit. A-rich vegetables*	12	37	187	38	194
*Other vegetables*	42	97	171	72	162
Fruits	44	99	90 ^b^	58	88
*Vit. A-rich fruits*	8	59	119	47	126
*Other fruits*	36	108	83	59	65
Fish and seafood	106	126	86 ^b^	41	78
Legumes	115	177	75 ^c^	11	77
Meat and poultry	118	211	57 ^d^	57	41
Starchy roots, and tubers	89	157	50 ^d^	28	47
Beverages	12	53	44 ^d,e^	48	60
Soups and sauces	34	131	41 ^d^	30	35
Eggs	14	171	40 ^d^	8	40
Cereals	169	224	31 ^e^	24	33
Milk and its products	23	192	28 ^e^	25	29
Nuts and seeds	30	500	27 ^e^	20	28
Fats and oils	35	881	−8 ^f^	21	−8
Miscellaneous	7	288	−15 ^f^	27	−15
*p*			<0.001		

Mean, standard deviation, and median NRF9.3 scores per 100 kcal by WAFCT food group. SD: standard deviation. ^1^ Means annotated with same-letter superscripts are not significantly different from each other. Subgroups of vegetables and fruits are italicized to indicate their inclusion within the broader vegetable and fruit categories. Values were rounded to the nearest integer.

**Table 3:** NRF6.3 scores by WAFCT food group per 100 kcal.

WAFCT Food Groups	No. of Foods	Kcal/100 g	Mean NRF6.3 Scores ^1^		
			Mean ^1^	SD	Median
Vegetables	113	73	139 ^a^	92	121
*Dark green leafy vegetables*	59	62	202	76	206
*Other vit. A-rich vegetables*	12	37	80	33	65
*Other vegetables*	42	97	67	52	52
Fish and seafood	106	126	107 ^b^	56	111
Meat and poultry	118	211	90 ^c^	100	61
Eggs	14	171	79 ^c^	35	67
Legumes	115	177	37 ^d^	13	36
Soups and sauces	34	131	37 ^d,e^	37	34
Milk and its products	23	192	29 ^e,f,j^	27	26
Cereals	169	224	20 ^f,j^	22	16
Fruits	44	99	19 ^f^	24	20
*Vit. A-rich fruits*	8	59	45	16	43
*Other fruits*	36	108	13	22	12
Starchy roots, and tubers	89	157	17 ^f,j^	22	14
Nuts and seeds	30	500	6 ^g^	17	7
Beverages	12	53	6 ^g,j^	50	−4
Fats and oils	35	881	−10 ^h^	18	−8
Miscellaneous	7	288	−27 ^i^	27	−28
*p*			<0.001		

Mean, standard deviation, and median NRF6.3 scores per 100 kcal by WAFCT food groups. SD: standard deviation. ^1^ Means annotated with same-letter superscripts are not significantly different from each other. Subgroups of vegetables and fruits are italicized to indicate their inclusion within the broader vegetable and fruit categories. Values are rounded to the nearest integer.

**Table 4:** NRF15.3 scores by WAFCT food group per 100 kcal.

WAFCT Food Groups	No. of Foods	Kcal/100 g	Mean NRF15.3 Scores		
			Mean ^1^	SD	NRF15.3
Vegetables	113	73	344 ^a^	179	296
*Dark green leafy vegetables*	59	62	452	163	446
*Other vit. A-rich vegetables*	12	37	251	55	266
*Other vegetables*	42	97	220	116	219
Fish and seafood	106	126	214 ^b^	73	211
Meat and poultry	118	211	196 ^b,c^	160	151
Eggs	14	171	138 ^c,d,j^	32	126
Fruits	44	99	107 ^d,e,j^	74	98
*Vit. A-rich fruits*	8	59	148	42	154
*Other fruits*	36	108	98	76	83
Soups and sauces	34	131	101 ^e,j^	47	94
Beverages	12	53	91 ^j^	90	88
Legumes	115	177	88 ^f,j^	21	88
Milk and its products	23	192	73 ^g,j^	47	66
Nuts and seeds	30	500	59 ^g,h,j^	33	69
Starchy roots, and tubers	89	157	58 ^g,h^	30	52
Cereals	169	224	50 ^h^	34	47
Fats and oils	35	881	12 ^i^	33	12
Miscellaneous	7	288	−11 ^i^	32	−14
*p*			<0.001		

Mean, standard deviation, and median NRF15.3 scores per 100 kcal by WAFCT food group. SD: standard deviation. ^1^ Means annotated with same-letter superscripts are not significantly different from each other. Subgroups of vegetables and fruits are italicized to indicate their inclusion within the broader vegetable and fruit categories. Values are rounded to the nearest integer.

**Table 5:** List of top 20 foods with the highest NRF scores *.

Ranking	Highest NRF9.3 Scores	Highest NRF6.3 Scores	Highest NRF15.3 Scores
1	Amaranth, leaves, raw	Chicken liver, raw	Spinach, leaves, raw
2	Amaranth, leaves, boiled	Chicken liver, stewed	Cowpea, leaves, raw
3	Spinach, leaves, raw	Chicken liver, boiled	Spinach, leaves, boiled
4	Spider plant, leaves, raw	Chicken liver, grilled (without salt or fat)	Cowpea, leaves, boiled
5	Spinach, leaves, boiled	Spider plant, leaves, raw	Spider plant, leaves, raw
6	Cowpea, leaves, raw	Spider plant, leaves, boiled	Spider plant, leaves, boiled
7	Spider plant, leaves, boiled	Amaranth, leaves, raw	Chicken liver, raw
8	Jute mallow (bush-okra), leaves, raw	Chicken giblets, grilled (without salt or fat)	Amaranth, leaves, fresh, raw
9	Mint, leaves, raw	Beef liver, raw	Jute mallow (bush-okra), leaves, raw
10	Cowpea, leaves, boiled	Mint, leaves, raw	Chicken liver, stewed
11	Moringa (drumstick), leaves, boiled	Chicken giblets, raw	Amaranth, leaves, boiled
12	Moringa (drumstick), leaves, raw	Amaranth, leaves, fresh, boiled	Chicken liver, grilled (without salt or fat)
13	Jute mallow (bush-okra), leaves, boiled	Eggplant, leaves, raw	Moringa (drumstick), leaves, raw
14	Eggplant, leaves, raw	Spider plant, leaves, boiled	Moringa (drumstick), leaves, boiled
15	Eggplant, leaves, boiled	Cowpea, leaves, raw	Beef liver, raw
16	Green leafy vegetable, average, raw	Beef liver, grilled (without salt or fat)	Chicken liver, boiled (without salt)
17	Pumpkin, leaves, raw	Beef liver, stewed	Spinach, leaves, boiled
18	Moringa (drumstick), leaves, boiled (without salt)	Chicken giblets, boiled	Eggplant, leaves, raw
19	Parsley, raw	Spinach, leaves, raw	Cowpea, leaves, fresh, boiled
20	Pumpkin, leaves, boiled	Mola carplet, small whole fish, raw	Beef liver, stewed

* Foods highlighted in green are classified as African indigenous vegetables (AIVs) and foods highlighted in blue are animal-source foods.

**Table 6:** Nutrient density of African indigenous vegetables (AIVs), non-indigenous vegetables, African indigenous grains (AIGs), and non-indigenous grains.

African Indigenous Food Groups	No. of Foods	Kcal/100 g	Mean (SD) NRF Score			Protein g/100 g Mean (SD)
			NRF9.3	NRF6.3	NRF15.3	
African indigenous vegetables (AIV)	25	46	339 (111)	198 (85)	451 (168)	3.8 (1.8)
Preparations with AIVs	18	73	228 (187)	133 (108)	324 (234)	4.2 (1.8)
Non-indigenous vegetables	68	54	227 (110)	111 (83)	292 (158)	2.9 (2.1)
*p*-value			<0.001 *	<0.001 *	0.001 *	<0.001 *
African indigenous grains (AIGs)	55	260	43 (19)	29 (11)	62 (20)	—
Non-indigenous grains	55	278	30 (21)	23 (25)	56 (43)	—
*p*-value		0.60	0.001 *	0.33	0.42	—
Non-indigenous grains (without fortified flours)	45	261	30 (23)	13 (8)	44 (32)	—
*p*-value **		0.97	0.002 *	<0.001 *	0.001 *	—

* Difference was statistically significant. NRF: Nutrient-Rich Food Index. SD: standard deviation. ** *p*-values of the comparison between AIGs and non-indigenous grains (without fortified flours). NRF scores are rounded to the nearest integer.

## Data Availability

Publicly available datasets were analyzed in this study. These data can be found at: https://www.fao.org/3/ca7779b/CA7779B.PDF (accessed on 26 February 2024). The original contributions presented in this study are included in the article; further inquiries can be directed to the corresponding author.

## Appendix A

**Table A1:** Food and beverage examples included in each food group.

Food Group	Examples
Vegetables	Cabbage, carrot, eggplant, onion, pepper, tomato, wild spinach
African indigenous vegetables	Amaranth leaves, spider plant, jute mallow, cowpea leaves, native eggplant, pumpkin leaves, moringa, sweet potato leaves, okra leaves
Cereals (non-indigenous grains)	Maize, wheat, rice, oats, bread
African indigenous grains	Fonio, pearl millet, teff, sorghum, native rice
Fish and seafood	African carp, anchovy, Atlantic cod, barracuda, bayad, catfish, mackerel, mahi mahi, mola carpet, perch, shrimp, tilapia, tuna
Meat and poultry	Beef kidney, beef liver, beef meat, camel meat, chicken giblets, chicken liver, chicken meat, game meat, goat meat, lamb meat, ostrich meat, pork meat, rabbit meat
Eggs	Chicken eggs, duck eggs, quail eggs, turkey eggs
Fruits	Apple, avocado, African black plum, banana, dates, figs, guava, mango, Orange, pineapple, tamarind, watermelon
Soups and sauces	Curry sauce with beef meat, fish and vegetable soup, clear sauce with fish, chicken soup
Beverages	Fruit juice, ovaltine, fruit nectar, soybean milk, teas
Legumes	African locust bean, Bambara groundnut, white beans, cowpeas, ground beans, lentils, soya beans
Milk and its products	Cow milk, cheese, camel milk, yoghurt
Nuts and seeds	Cashew nut, cola nut, bitter cola seeds, groundnut, hibiscus seed, melon seeds, pumpkin seeds, sesame seeds
Starchy roots, and tubers	Cassava, cocoyam, plantain, sweet potato, potato, water yam
Fats and oils	Butter, coconut oil, corn oil, cottonseed oil, groundnut oil, margarine, palm oil, soya oil, sunflower oil, vegetable oil
Miscellaneous	Honey, jam, sugar, sweets, vinegar
